# Gutta-Percha as a Filling-Material

**Published:** 1894-11

**Authors:** Arthur H. Stoddard

**Affiliations:** Boston


### GUTTA-PERCHA AS A FILLING-MATERIAL.[1]

BY ARTHUR H. STODDARD, D.M.D., BOSTON.

It is now many years since J. Foster Flagg announced this "new departure" in filling, and although most of us believe that his ground in the matter is extreme, yet there is an element of truth in his theory which every unprejudiced man should accept.

Most reformers are likely to be extremists, and it is only after the lapse of time that their theories settle to their proper place.

We recognize this in "the mind-cure and other pseudo-science," of which we hear from time to time, and, although we are not prepared to accept the theories as a whole, yet we cannot deny there is an element of truth in them. So it is with the "new departure."

---

[1] Read before the Harvard Odontological Society, May 31, 1894.

There are certain cavities and conditions of the teeth where gutta-percha is unquestionably the best, as in other cases it might be the worst, material that could be used in filling. As a temporary filling in treating dead teeth, stopping canals, separating, etc., its value is acknowledged by all. But I wish to call your attention to-night particularly to its value as a permanent stopping. It is no longer a theory, but a fact proved by experience, that I have been able to save many teeth by its use which could not have been saved by any other means. I refer particularly to very frail teeth of persons who, through neglect or improper treatment, have large cavities in the buccal or approximal surfaces of the molars and bicuspids. I may add, however, that in cases where they are subject to the wear of mastication, I almost always cap with oxyphosphate of zinc, but I usually prefer to fill without cutting down from the grinding surface when the cavity can be properly excavated without. Many times shallow cup-shaped approximal cavities without undercut can be filled with gutta percha, when it would be impossible to insert gold or amalgam without cutting away considerable sound dentine.

In cases where I insert cement filling on approximal surfaces, I always build in the cervical wall with gutta-percha. This overcomes the liability to disintegration of the cement. I generally use gutta-percha in approximal surfaces in children's teeth, and sometimes in the fissures in molars, if for any reason cement or tin are excluded. In this manner the teeth can be kept along for years, till they become hard enough for gold or amalgam fillings.

The durability of gutta-percha fillings depends on the kind of gutta-percha. The method of manipulation, the manner of heating, the location in the mouth, the location in tooth, and on the secretions of the mouth. In some mouths the surface of the gutta-percha decomposes to some extent. I recall one case where the surface of the gutta-percha became reddish in color, due no doubt to a fungus growth. When this is removed it remains white but a short time, and then assumes the reddish color again. It is now over three years since I first saw these fillings, and they are still in good condition.

The success of gutta-percha fillings depends greatly on their manipulation. The manner of heating is of the greatest importance. If heated so that it swells or blisters, it is ruined and should be thrown away. It should not be heated in an open flame, as the angles of the pellet become burned while the interior is scarcely warm. I use a metal or soapstone disk high enough above the flame to allow it to remain at about the necessary temperature for heating the gutta-percha, while the instruments can be heated to best advantage in the open flame, as they should be considerably warmer than the gutta-percha. It is advisable to wipe out the cavity with resin varnish and insert the gutta-percha in small pellets; when a little more than full condense with a warm flat instrument; after having carefully trimmed overhanging edges, smooth with linen tape slightly saturated with oil of cajuput.

The best gutta-percha I have found is "dentron." It is durable, packs well under a warm instrument, is not elastic, and does not drag, although it requires considerable heat to soften it. Almost all the gutta-percha I have tried, except this, has a tendency to drag from the wall of the cavity unless manipulated with a very warm instrument.

The gutta-percha which I shall pass around I prepare myself, and, although it is intended principally as a temporary filling, yet I use it occasionally as permanent filling in such places as the lingual surfaces of the lower molars. It softens at low heat, packs readily in the cavity, and does not drag; and it becomes quite hard after it has been in the mouth for some time. It is prepared from Knapp's sheet gutta-percha, is cut into strips, and allowed to stand two or three days in a solution of oil of cajuput and chloroform, equal parts. Then it is warmed till it becomes thoroughly soft, and plaster of Paris is added till it becomes about the consistency of putty. It may then be taken into the hands and kneaded thoroughly, rolled into strips, and allowed to remain in the open air till the chloroform and cajuput evaporates, when it is ready for use.

